# Explainable Hybrid Deep‐Radiomic Graph Learning Framework for Automated IDC Aggressiveness Grading From Whole‐Slide Histopathology Images

**DOI:** 10.1155/ijbi/7498792

**Published:** 2026-11-10

**Authors:** Vijayalaxmi Inamdar, Shaila S. G.

**Affiliations:** ^1^ Dayananda Sagar Academy of Technology and Management Bengaluru Karnataka India dayanandasagar.edu; ^2^ School of Engineering Dayananda Sagar University Bengaluru Karnataka India

**Keywords:** breast cancer, deep-radiomic model, dense conditional random field, graph attention network, graph neural network, histopathology, invasive ductal carcinoma, multiple instance learning

## Abstract

Breast cancer remains one of the leading causes of cancer‐related mortality among women worldwide. Histopathological analysis of whole‐slide images (WSIs) plays a crucial role in the diagnosis and grading of breast cancer subtypes. Invasive ductal carcinoma (IDC) is the most prevalent and heterogeneous subtype of breast cancer, and its aggressiveness grading has important prognostic value. Manual grading of IDC aggressiveness using hematoxylin and eosin (H&E) stained WSIs is tedious and subjective. In this study, we address the issues of automated IDC aggressiveness grading using an explainable hybrid deep‐radiomic graph learning framework. The proposed pipeline jointly performs tumor segmentation, feature representation, and classification within an end‐to‐end framework. The tumor is segmented using an attention U‐Net followed by dense conditional random field (DenseCRF) segmentation refinement. Deep convolutional features and radiomic features encoding texture, morphology, and intensity statistics are extracted from the segmented tumor region and combined to form hybrid deep‐radiomic features. Spatial graphs constructed from these hybrid features are subsequently processed using a graph attention network (GAT)–based graph neural network (GNN) with attention‐based multiple instance learning (MIL) to reason about the relationships between different patches for slide‐level grade prediction. Extensive experiments performed on two datasets, BreakHis and BACH, show that our proposed architecture demonstrates improved performance in IDC aggressiveness grading when compared with convolutional neural network (CNN) and GNN baselines with an accuracy of 98.3%, macro‐F1 score of 0.968, ROC‐AUC score of 0.98, and Dice score of 0.932. The explainability of proposed model with Grad‐CAM visual explanations, graph attention scores, and SHAP‐based feature importance scores for individual radiomic features are presented.

## 1. Introduction

Breast cancer is one of the most frequent causes of cancer‐related death in females globally. The most current global burden estimates from GLOBOCAN 2024 were recently published by the International Agency for Research on Cancer (IARC) and the American Cancer Society (ACS) [1, 2]. Among different types of breast cancer, invasive ductal carcinoma (IDC) constitutes about 70%–80% of all cases [3]. Morphologically diverse and clinically heterogeneous, precise grading of IDC aggressiveness guides therapy planning, prognostic grouping, and customized treatment decision‐making. Pathologists assess the aggressiveness of IDC by visually examining hematoxylin and eosin (H&E) stained whole‐slide images (WSIs) as the current clinical practice [4]. However, manual grading of breast cancer is laborious and subjective, often leading to interobserver variability especially in ambiguous and morphologically heterogeneous cases. Objective, reproducible, and scalable computational solutions are therefore required to aid pathologists in grading IDC aggressiveness [5]. Recent developments in artificial intelligence (AI)–based computational pathology approaches have shown promise for automating breast cancer analysis tasks [6]. Deep learning methods automatically learn the discriminative visual patterns from histopathology images for detecting and classifying tumors [7]. On the other hand, radiomics‐based approaches model quantitative descriptors that encode interpretable information about tissue morphology, texture, and intensity distributions [8]. Most of these studies rely on independent modalities either learning deep features or utilizing handcrafted radiomic features. The incorporation of these rich descriptors can achieve more complete characterization of tumor heterogeneity; however, deep learning methods are often considered “black‐box” models that provide little insight into their outputs. Although these techniques advance the task, many important limitations remain such as

a. weak representation of interpatch spatial correlations at slide‐scale;
b. poor generalizability across datasets due to staining/acquisition variability;
c. class imbalance; and
d. absence of cohesive, hierarchical explainability for clinical interpretability.

Motivated by the above research gaps, this work proposes explainable hybrid deep‐radiomic graph learning framework for automated IDC aggressiveness grading from WSI. Unlike existing hybrid deep learning and graph‐based approaches that address only individual aspects of feature representation, contextual learning, or explainability, the proposed framework integrates attention‐guided tumor segmentation with dense conditional random field (DenseCRF) refinement, adaptive fusion of complementary deep and radiomic features [9], graph neural network (GNN)‐based spatial contextual reasoning [10], attention‐based multiple instance learning (MIL) [11], and multilevel explainability within a unified end‐to‐end architecture. By jointly modeling tumor morphology, quantitative radiomic characteristics, and interpatch spatial relationships, the developed framework captures both local and global tissue heterogeneity more effectively. Furthermore, the integration of gradient‐weighted class activation mapping (Grad‐CAM) [12], graph attention visualization, and SHapley Additive exPlanations (SHAP)–based[13] feature attribution provides comprehensive explainability at the image, graph, and feature levels, thereby improving the transparency and clinical applicability of automated IDC aggressiveness grading. The contributions of the proposed framework are summarized as follows:

a. End‐to‐end hybrid deep‐radiomic pipeline: A unified end‐to‐end deep‐radiomic learning pipeline that jointly performs tumor segmentation, feature extraction, and graph‐based grading classification tasks for automated IDC aggressiveness grading is performed.
b. Attention‐guided segmentation: An attention‐guided U‐Net combined with DenseCRF‐based postprocessing enables precise segmentation of tumor regions from heterogeneous histopathological images.
c. Dual‐stream feature representation and adaptive fusion: Leveraging handcrafted radiomic features and deep convolutional embeddings through an adaptive fusion scheme allows proposed approach to exploit both low‐level tissue characteristics and high‐level semantic features.
d. Graph attention learning with GNN‐MIL: Building a graph with patch embeddings that models both spatial adjacency and feature‐based relationships, followed by contextual dependencies with GNN and aggregating them with attention‐based MIL allows learning tissue relationships and their spatial interactions to derive slide‐level IDC aggressiveness grades.
e. Multilevel explainability for clinical transparency: The integration of Grad‐CAM, GNN/MIL attention maps, and SHAP‐based radiomic feature attribution provides multilevel explainability, enhancing the transparency and clinical reliability of the proposed hybrid deep‐radiomic model.
f. Validation and generalization analysis: The proposed approach is exhaustively evaluated on public Breast Cancer Histopathological Database (BreakHis) and BreAst Cancer Histology (BACH) datasets to show improved performance with respect to accuracy, robustness, calibration, and crossdataset generalizability.

The remainder of this paper is organized as follows. Section 2 presents the literature review. Section 3 describes the proposed hybrid deep‐radiomic graph–based framework for IDC aggressiveness grading. Section 4 presents the experimental setup, performance evaluation, and discussion of the results, including ablation and computational analyses. Finally, Section 5 concludes the paper by summarizing the key findings, limitations, and future research directions.

## 2. Literature Survey

Many AI‐based computational pathology approaches have been recently developed to automate breast cancer diagnosis and grading ([13–16]. Existing literature can be roughly divided into four categories: conventional deep learning–based classification algorithms, hybrid deep learning–radiomics methods, graph‐based/context‐aware learning methods, and explainable AI (XAI) algorithms for better clinical explainability. Representative studies belonging to each category are reviewed below from critical aspects such as their contributions, limitations, and existing gaps. These gaps are further motivated to develop explainable an hybrid deep‐radiomic graph learning framework, which is discussed in this paper.

### 2.1. Deep Learning Techniques

Broad spectrum of computational pathology and medical image analysis algorithms have been developed over the past few years that is aimed at automatically detecting and assessing aggressiveness grade of IDC. In recent years, there has been significant interest in using radiomics, deep learning, and computer‐aided diagnostic algorithms to objectively and reproducibly grade IDCs. Traditional histopathological analysis, which remains the clinical gold standard for IDC detection and grading, suffers from subjective assessment and observer bias which makes it nonscalable and susceptible to interobserver variability. Hybrid tumor segmentation/radiomic feature extraction or ensemble‐based learning/deep learning–based classification frameworks have also been explored to overcome shortcomings in previous studies. The recent work presents survey involving tumor detection, segmentation, feature fusion, and grading to highlight common trends, shortfalls, and areas of future work required to engineer accurate, scalable, and interpretable fully automated IDC grading pipelines. The work in this section presents convolutional neural networks (CNNs), transfer learning, transformers, or deep neural networks in general without explicitly combining radiomics or graph learning. Farooqui and Ritika [17] explored machine learning techniques that aid early detection and prevention of breast cancer. The authors highlighted the promise of data‐driven methodologies to assist clinical diagnosis. The studies mentioned in this section solidify the relevance of machine learning and deep learning approaches to healthcare analytics and motivate need for more accurate, interpretable frameworks that can be trusted by clinicians. Ayana et al. [18] introduce a multistage transfer learning framework for ultrasound breast cancer image classification. The proposed method fine‐tunes transfer learning weights progressively and demonstrates improved performance over the existing modalities. However, while reducing model training overhead, this method exclusively uses deep learned features without investigating handcrafted or radiomic features. Furthermore, contextual or spatial relationships between tissues regions are not modeled. Jabeen et al. [19] proposed an optimal probability‐based deep feature fusion approach to classify breast cancer from ultrasound images. A fusion strategy is proposed that optimizes feature vectors from diverse pretrained networks for better discrimination. Although their results show improvement over individual feature vectors from respective networks, the study only formulates breast cancer detection as a binary classification task. Assessment of tumor aggressiveness is not considered. In addition, explainability methods are not investigated which limits clinical explainability. Nehary and Rajan [20] studied an ensemble of deep learning classifiers for ultrasound breast image classification and report improvement in overall accuracy over individual classifiers. This work increases model robustness to noise by leveraging predictions from an ensemble of classifiers. However, segmentation masks are not used to guide feature extraction which could introduce background context into feature descriptions. Misra et al. [21] study a bimodal transfer learning approach using B‐mode ultrasound images and strain images of breasts to classify cancerous regions. Both imaging modalities provide information about the structural and elasticity distribution of the breast, therefore improving classification performance. Alotaibi et al. [22] proposed a vision transformer and the data‐efficient image transformer **(**ViT–DeiT) ensemble learning framework for breast cancer classification from histopathological images. The ViT‐based design enables better learning of long‐range spatial dependencies and achieves better results than conventional CNN‐backboned classifiers. Drioua et al. [23] present an overview of deep learning–based segmentation methods for breast cancer histopathology images. By applying existing segmentation models, the authors show improvement in tumor cell segmentation from normal tissue when there are variabilities in staining procedures. This study solely focuses on segmentation and does not utilize these segmented regions for downstream classification tasks. There is no discussion of post segmentation analysis pipelines which would be required for clinical implementation. There exists an absence of strategies for addressing interslide variability and the lack of discussion on model explainability. Lamprou et al. [24] designed StethoNet, which is an ensemble‐based deep learning framework designed to classify breast cancer from mammography images. The classifier was more robust to noisy imaging and acquisition artifacts and showed strong breast cancer classification performance on publicly available datasets. Madhu et al. [25] designed UCapsNet, a two‐stage deep learning architecture based on U‐Net–segmentation followed by capsule networks. Their deep learning framework leveraged scan path‐based routing and showed improved stability to rotation and geometric effects in ultrasound images. Their method improves detection of lesion boundaries and shows promising breast cancer classification performance. Ramamoorthy et al. [26] introduced a deep learning–based method for predicting breast cancer presence using histopathology WSIs. The framework was applied to multiple CNN models and showed accuracy comparable with similar state‐of‐the‐art methods. Recent advances in computational pathology have demonstrated the effectiveness of deep learning for automated breast cancer diagnosis using histopathological images. Ding et al. [27] proposed a multibranch attention network for breast cancer histopathology classification, achieving improved feature representation through attention‐guided learning. Jaganathan et al. [28] employed transfer learning strategies to improve diagnostic performance. Alharbi et al. [29] introduced a novel 59‐layer dense inception network for robust deepfake identification, highlighting the effectiveness of deep hierarchical architectures in learning complex feature representations from large‐scale datasets. Although originally proposed for deepfake detection, the study demonstrates the effectiveness of deep hierarchical architectures for learning highly discriminative visual representations.

Collectively, these deep learning approaches have significantly improved breast cancer detection and classification by learning discriminative visual representations directly from medical images. Nevertheless, most methods perform independent patch‐ or image‐level analysis and rely solely on deep feature representations, limiting their ability to explicitly model tissue heterogeneity, interpatch contextual relationships, and clinically interpretable biomarkers. Furthermore, explainability and robustness across heterogeneous datasets remain insufficiently addressed, highlighting the need for more comprehensive and clinically transparent frameworks.

### 2.2. Hybrid Deep‐Radiomic Methods

This section presents studies combining handcrafted features and deep learning. Amrisha et al. [30] demonstrated breast cancer detection using deep learning with two different imaging modalities. By using two complementary sources of information about breast cancer, their model performed better than using just one imaging modality. Although their results are promising toward multimodal deep learning, fusion of features is performed at image level without considering an integration scheme to combine complimentary information from each modality. Srikantamurthy et al. [31] introduced HistoNet, a hybrid CNN–LSTM (long short‐term memory)–based transfer learning framework to classify benign and malignant breast histopathology images. The fusion of CNN and LSTM allows the model to not only learn spatial representations of the tumor cell patterns but also sequential dependencies between patches to improve classification accuracy. Because there is no clear temporal relationship between histopathology patches within a slide, modeling long‐range dependencies using LSTM does not conceptually fit the problem. Molaei et al. [32] developed a hybrid framework for cancer detection using particle swarm optimization and feedforward artificial neural networks (ANN) to detect cancer using microRNA biomarkers. Particle swarm optimization resulted in better selected features and more stable convergence in training the ANN. Their method demonstrates improved performance over several benchmark machine learning methods when tested on available tabular data. Thatha et al. [33] proposed a deep learning–based breast cancer diagnosis method using histopathology images of breast cancer tissues. Fusion of handcrafted and deep learning features using K‐nearest neighbor (KNN) resulted in stable and accurate breast cancer classification. Their method performs better comparably with other state‐of‐the‐art works. However, this method only works for binary classification of breast cancer. Integration of the moth‐flame optimization module results in additional computational complexity. The method does not model any spatial dependence between nearby patches of tissue in the WSI.

Overall, hybrid deep‐radiomic approaches have demonstrated that combining handcrafted radiomic descriptors with deep learning representations improves classification performance by capturing complementary morphological and quantitative tissue characteristics. However, existing methods predominantly focus on feature‐level fusion and do not explicitly incorporate spatial contextual reasoning among tissue regions. Moreover, most studies formulate binary classification problems and provide limited explainability, restricting their applicability to automated IDC aggressiveness grading.

### 2.3. Graph‐Based Learning and Context‐Aware Frameworks

This section focuses on graph learning, weak supervision, and contextual reasoning. Paul et al. [34] presented a weakly supervised deep learning framework that can predict survival outcomes from histopathology WSIs of breast carcinoma. Patch‐level representations are pooled to create a robust slide‐level representation for outcome prediction. The proposed method identifies morphological features relevant to breast cancer survival outcome prediction from breast carcinoma WSIs. Iqbal and Sharif [35] proposed breast tumor‐Swin transformer (BTS‐ST), a two‐stage framework based on Swin transformer for segmentation and classification of histopathology images from multimodal breast cancer imaging data. The Swin transformer captures long‐range information within and across modalities and therefore achieved better results than competitive baselines. Although the authors achieve strong results, the training time and computational resources required for Swin transformers makes it difficult to train these models without access to large‐scale datasets. Chan et al. [14, 16] proposed a heterogeneous graph representation learning framework for WSI analysis by modeling different nucleus types and their semantic interactions within a heterogeneous graph. Their framework utilized a heterogeneous graph edge attribute transformer (HEAT) to capture complex cellular relationships and introduced semantic‐consistent pooling for graph‐level representation learning. Experimental evaluation on multiple “The Cancer Genome Atlas (TCGA)” datasets demonstrated improved classification performance and enhanced explainability through node‐level attribution. However, the framework mainly depends on graphs generated from cellular interactions without handcrafted radiomics features to supplement the graph data. Shi et al. [36] proposed a graph‐transformer framework with graph convolutional networks and transformer‐based attention mechanism for weakly supervised classification of WSIs. Graph transformer integration (GTI) module effectively captures local spatial correlations between adjacent patches and global context information of long‐range dependencies. The framework demonstrates improved performance over traditional attention‐based MIL approaches across several public WSI datasets. Although the framework effectively captures multiscale contextual information, it relies exclusively on deep feature embeddings and does not exploit interpretable radiomic biomarkers or multilevel explainability to support clinically transparent decision‐making. Ding et al. [37] introduced a framework called dynamic graph and state‐space model–based MIL (DG‐SSM‐MIL) for WSI classification. Graph attention networks (GAT), combined with state‐space modeling, are leveraged to learn local spatial dependencies and global context simultaneously and achieved state‐of‐the‐art results on multiple public WSI datasets. However, this framework only aimed at reasoning contextual information via graphs without directly incorporating deep handcrafted radiomic features, attention‐based tumor segmentation, and multilevel explainability. More recently, Zhao and Du [38] introduced a graph‐assisted global reasoning framework that captures contextual tissue relationships for breast cancer histopathological image classification. The findings are consistent with recent graph‐based and attention‐driven computational pathology studies that have demonstrated the importance of contextual tissue modeling and attention‐guided feature learning for breast cancer histopathological image analysis.

Thus, recent graph‐based computational pathology frameworks have demonstrated the effectiveness of GNNs, graph transformers, and attention‐based MIL in modeling contextual dependencies among histopathological tissue regions, thereby improving slide‐level representation learning and classification compared with conventional patchwise approaches. Despite these advances, existing methods predominantly rely on deep feature embeddings and provide limited integration of complementary radiomic biomarkers, attention‐guided tumor segmentation, and comprehensive multilevel explainability within a unified framework. Consequently, there remains a significant research gap in developing an end‐to‐end, explainable framework that jointly exploits morphological, quantitative, and spatial tissue characteristics for robust and clinically interpretable IDC aggressiveness grading. This research gap forms the primary motivation for the proposed explainable hybrid deep‐radiomic graph learning framework presented in this work.

### 2.4. XAI in Histopathology

XAI has recently gained significant attention in computational pathology to improve the transparency and clinical reliability of deep learning models. Visual explainability methods like Grad‐CAM offer region‐level explanations through the visualization of diagnostically relevant regions of interest (tissue), whereas feature attribution techniques like SHAP analyze the impact of specific handcrafted features on model output. Visualization of attention mechanisms has also been used to interpret the learned contextual relationships in GNNs and MIL. Li et al. [39] presented an AI‐assisted diagnostic system for breast cancer detection and treatment planning for use in low‐resource countries. Authors present their aim of creating a transparent, interoperable workflow that leveraged artificial intelligence to detect breast cancer. This algorithm has the potential to serve as a decision support tool for diagnosing breast cancer earlier. Jiang et al. [40] recently published a review of deep learning–based AI for breast cancer histopathology. Hosny et al. [41] discussed recent XAI methods for medical image analysis and classification, emphasizing the emergence of Grad‐CAM, SHAP, and LIME (local interpretable model‐agnostic explanations) as common techniques. Their survey revealed that when performing multiple explanation methods together, it gave clinicians better visuals as well as feature‐level explanations which helped supplement one another and increase trust when using AI for diagnosis. However, they noted there is still no standard explainability solutions that can offer region‐level, feature‐level, and context‐level explanations for deep visual recognition tasks in medical imaging. Houssein et al. [42] conducted a review of XAI in medical imaging and summarized the current approaches into four categories: saliency‐based explanations, attention‐based explanations, model‐agnostic feature attributions, and interpretable models. The study concluded that, although visualization techniques such as Grad‐CAM can effectively localize diagnostically relevant regions, important challenges remain regarding the systematic evaluation, robustness, interpretability, and clinical validation of these explanation techniques. Abbas et al. [43] discussed the importance of explainable machine learning in clinical applications, stating that transparency and explainability will be required for AI‐based decision support to gain widespread clinical adoption. The authors claim that XAI will increase physicians′ trust in the technology, allow more rigorous model validation, and promote responsible clinical decision‐making by allowing users to understand how the model arrived at a given prediction. Suara et al. [44] presented the use of Grad‐CAM for interpreting medical images. The authors concluded that saliency‐based visualization increases the explainability of deep learning models by allowing users to identify areas of the input image that were significant to the model′s prediction. The study also found that Grad‐CAM explanations were not always considered reliable or complete, indicating a need for additional explainability methods to increase clinicians′ trust in AI‐generated predictions. Ahmed et al. [45] introduced a fusion‐based machine learning model for stroke risk prediction demonstrating that the integration of multiple learning models can improve predictive accuracy and clinical decision support.

Even though XAI has become an integral aspect of computational pathology aiding the reliability, interpretability, and clinical trust of deep learning algorithms, the majority of recent studies focus on using a single explanation technique where image‐level or feature‐level interpretations are provided separately. Integrated multilevel explainability methods that leverage visual, feature, and contextual interpretation simultaneously are needed.

Table 1 compares proposed hybrid deep‐radiomic model to recent state‐of‐the‐art hybrid deep learning and graph‐based IDC grading methods. This comparison allows us to highlight each framework′s capabilities and recognize the contributions necessary to address important research gaps.

**Table 1:** Comparative analysis of state‐of‐the‐art hybrid deep learning and graph‐based IDC grading frameworks.

Approaches	Attention‐guided segmentation	Deep‐radiomic fusion	Graph‐based context modeling	Attn‐based MIL	Multilevel explainability	IDC aggressiveness grading
Ayana et al. [18]	No	Deep features	No	No	No	No
Iqbal and Sharif [35]	Yes	Deep features	Transformer context	No	Limited	No
Alotaibi et al. [22]	No	Deep features	Implicit (ViT)	No	No	No
Srikantamurthy et al. [31]	No	Partial	Sequential (LSTM)	No	No	Binary
Paul et al. [34]	No	Deep features	No	Yes	Limited	No
Thatha et al. [33]	No	Yes	No	No	No	Binary
Proposed framework	Yes	Yes	Yes (GNN)	Yes	Grad‐CAM + GNN attention + SHAP	Yes (multiclass)

Recent studies summarized in Table 1 have shown promising advancement toward deep learning, radiomics, graph learning, and attention approaches for breast cancer. However, most methods focus on solving single components of the IDC grading pipeline and rarely incorporate multiple, complementary techniques. Current hybrid radiomic and deep learning methods focus on early fusion of handcrafted radiomic descriptors and deep learning representations, whereas graph‐based methods concentrate on contextual reasoning through learned deep embeddings without exploiting interpretable radiomic biomarkers. Likewise, attention modules have been mostly utilized toward segmentation or weakly supervised aggregation tasks. Multilevel explainability remains insufficiently explored. Limitations of existing methods highlight that current solutions fail to offer a joint model capable of achieving accurate tumor localization and segmentation, learning complementary deep‐radiomic feature representations, explicit spatial contextual reasoning, attention‐based slide‐level prediction aggregation, and clinically interpretable decision‐making. Bridging these gaps, our proposed architecture presents an end‐to‐end explainable hybrid deep‐radiomic graph–based framework for automatic aggressiveness grading of IDC from histopathological WSIs. The detailed description of the proposed methodology is discussed in the below section.

## 3. Proposed Methodology

To address the above‐mentioned limitations, we propose an explainable hybrid deep‐radiomic graph–based framework for automatic aggressiveness grading of IDC from histopathological WSIs. The proposed pipeline includes attention‐based tumor segmentation followed by DenseCRF refinement, dual deep and radiomic feature extraction streams, adaptive multistream feature fusion with embedded dimensionality reduction, and graph‐based contextual reasoning using GAT‐based GNN. Finally, slide‐level predictions are obtained using attention‐based MIL. Additionally, we provide multilevel explanations using Grad‐CAM, graph attention maps, and SHAP‐based radiomic feature attribution.

### 3.1. Proposed Framework Architecture

The proposed system is an end‐to‐end computational pathology pipeline that processes WSIs and predicts IDC aggressiveness grades (low, intermediate, and high). The overall workflow is illustrated in Figure 1, which consists of following stages.

a. Preprocessing and tiling of WSIs.
b. Attention‐guided tumor segmentation.
c. Dual‐stream feature extraction.
d. Attention‐based feature fusion and graph construction.
e. GAT‐based GNN with MIL.
f. Slide‐level aggregation and IDC aggressiveness grading.
g. Explainability using Grad‐CAM, SHAP, and graph attention maps.

**Figure 1:**
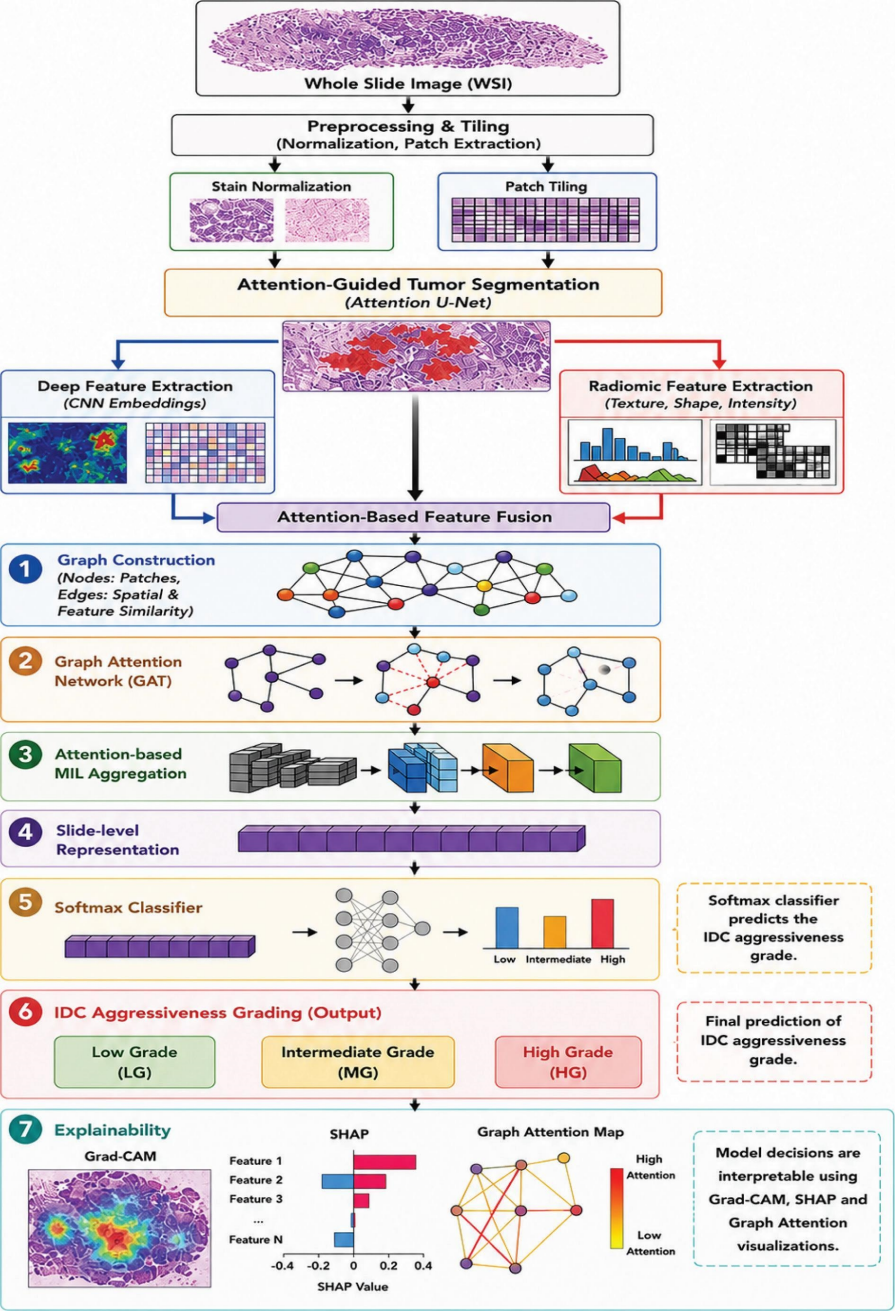
Architecture flow of proposed deep‐radiomic graph learning framework.

The proposed explainable hybrid deep‐radiomic graph learning framework performs automatic IDC aggressiveness grading from WSIs by integrating deep learning, radiomics, graph learning, and XAI. Initially, the WSI is preprocessed through stain normalization and patch tiling to reduce staining variability and generate manageable image patches. An attention U‐Net then segments the tumor regions, ensuring that only diagnostically relevant tissue is analyzed.

The segmented patches are subsequently processed through two parallel feature extraction branches such as CNN that extracts high‐level deep semantic features, and radiomic analysis computes handcrafted texture, shape, and intensity descriptors that capture clinically meaningful characteristics. These complementary features are adaptively combined using an attention‐based feature fusion module to produce a unified representation. A graph is then constructed where image patches serve as nodes and edges represent spatial proximity and feature similarity, enabling a GAT to model contextual relationships among tumor regions. The resulting graph features are aggregated using an attention‐based MIL mechanism to generate a discriminative slide‐level representation, which is subsequently classified by a softmax classifier into low‐grade (LG), intermediate‐grade (IG), or high‐grade (HG) IDC aggressiveness categories. To improve transparency and clinical interpretability, the framework incorporates Grad‐CAM for highlighting discriminative tumor regions, SHAP for quantifying the contribution of individual features, and graph attention maps for visualizing influential node interactions within the graph. By jointly leveraging complementary deep and radiomic features with graph‐based contextual learning and explainable AI techniques, the proposed framework provides accurate, robust, and clinically interpretable IDC aggressiveness grading.

The architectural components employed in the proposed hybrid deep‐radiomic model were carefully selected to address three major challenges in breast histopathological image analysis, namely, complex tissue morphology with ambiguous tumor boundaries, heterogeneous spatial organization of tissue regions, and the need for clinically interpretable decision‐making. The rationale for selecting each component over representative alternatives is summarized in Table 2.

**Table 2:** Rationale for selecting the major components of the proposed framework.

Component	Alternative models	Selected model	Justification
Tumor segmentation	U‐Net, U‐Net++, DeepLabV3+, TransUNet	Attention U‐Net	Employs attention gating to suppress irrelevant tissue regions while enhancing diagnostically significant tumor structures, thereby improving segmentation accuracy in heterogeneous histopathological images.
Boundary refinement	Morphological operations, fully connected CRF, no refinement	DenseCRF	Refines segmentation boundaries by enforcing spatial and appearance consistency, resulting in smoother and anatomically coherent tumor delineation.
Graph‐based contextual learning	GCN, GraphSAGE, GIN	GAT	Learns adaptive attention weights for neighboring tissue patches, enabling effective modeling of heterogeneous spatial interactions and contextual tissue dependencies compared with uniform neighborhood aggregation.

### 3.2. Preprocessing and Tiling

WSIs exhibit large resolution, staining variability, and background artifacts, necessitating standardized preprocessing. Initially, stain normalization is applied using Vahadane normalization [46] to reduce interslide color variation. Subsequently, nontissue regions, background areas, folds, and blurred regions are removed using thresholding and morphological filtering. The processed WSIs are then divided into smaller image patches *x*_*i*_ of size *T* × *T*, where *T* ∈ {256, 512}, with an optional overlap determined by stride *s*. Patch sizes of 256 × 256 and 512 × 512 were selected to balance computational efficiency with the preservation of local cellular morphology and tissue‐level contextual information. The spatial coordinates *p*_*i*_ = (*u*_*i*_, *v*_*i*_) of each patch are retained for subsequent graph construction. Each patch undergoes per‐channel *z*‐score normalization as represented in Equation (1) below.

(1) xi′=xi−μcσc,  ∀c∈R,G,B,

where *μ*_*c*_ and *σ*_*c*_ denote the mean and standard deviation of channel *c*.

This normalization reduces interpatch intensity variability, improves training stability, and facilitates consistent feature extraction across the entire WSI.

### 3.3. Tumor Segmentation

Accurate tumor segmentation is essential for reliable extraction of tumor‐specific morphological and radiomic features. Therefore, the proposed architecture employs Attention U‐Net [47] to segment tumor regions from histopathological image patches. The segmentation network was trained using expert‐annotated tumor masks from the BACH dataset, where each pixel was assigned a binary label indicating 1 for tumor pixel and 0 for background pixel. These annotations served as supervisory signals for pixelwise learning. Each extracted histopathological image patch was paired with its corresponding binary mask, which served as the ground‐truth annotation for pixelwise supervised learning.

The proposed segmentation module employs a 2D encoder–decoder Attention U‐Net architecture comprising four encoder (down‐sampling) blocks, a bottleneck layer, and four decoder (up‐sampling) blocks connected through attention‐guided skip connections. Each encoder block contains two consecutive 3 × 3 convolutional layers, each followed by batch normalization and ReLU activation, and a 2 × 2 max‐pooling operation for spatial down‐sampling. The bottleneck layer captures high‐level semantic representations before feature reconstruction in the decoder. Each decoder block performs 2 × 2 transposed convolutions for up‐sampling, followed by concatenation with the corresponding encoder features through skip connections and two additional 3 × 3 convolution–batch normalization–ReLU operations to progressively recover spatial resolution. Unlike the conventional U‐Net architecture, Attention U‐Net incorporates attention gates along each skip connection. These gates automatically suppress irrelevant background activations while selectively emphasizing diagnostically significant tumor regions before feature fusion in the decoder. Consequently, the network learns to focus on morphologically informative structures, including tumor boundaries, nuclei‐dense regions, and heterogeneous tissue patterns, thereby improving segmentation accuracy in complex histopathological images. The final decoder output is passed through a 1 × 1 convolutional layer followed by a sigmoid activation function to generate a pixelwise tumor probability map. Although Attention U‐Net provides accurate segmentation, tissue heterogeneity and staining variability may still produce minor boundary irregularities and isolated misclassified pixels. Therefore, DenseCRF [48] is employed as a postprocessing step to refine the predicted segmentation masks by jointly modeling spatial and appearance similarities among neighboring pixels. This refinement removes isolated misclassified pixels, sharpens tumor boundaries, and generates spatially coherent segmentation masks that preserve fine structural details for subsequent deep feature extraction and radiomic analysis.

The segmentation network is optimized using a hybrid loss that combines Dice loss and binary crossentropy (BCE) loss as represented in Equation (2).

(2) Lseg=λdiceLDice+λceLCE.

Here, *L*_seg_ denotes the overall segmentation loss used to optimize the attention‐guided U‐Net model. *L*_Dice_ and *L*_CE_ represents Dice loss and BCE loss, respectively. *λ*_dice_ and *λ*_ce_ are nonnegative hyperparameters. *L*_Dice_ measures the overlap between the predicted segmentation mask and the ground‐truth mask. It focuses more on region‐level consistency between the tumor and background pixels, which is useful when dealing with class imbalance. *L*_CE_ penalizes pixelwise classification errors and tries to model the probability output of each pixel. *L*_Dice_ and *L*_CE_ are computed as shown in Equations (3) and (4).

(3) Dice loss LDice=1−2∑p=1Pypyp′+ϵ∑p=1Pyp+∑p=1Pyp′+ϵ,

(4) Binary crossentropy lossLCE=1P∑p=1Pyplogyp′+1−yplog1−yp′,

where *y*_*p*_ ∈ {0, 1} is the ground‐truth label at pixel *p*, *y*′_*p*_ ∈ [0, 1] is the predicted probability at pixel *p*, *P* is the total number of pixels in the patch, and *ϵ* is the smoothing constant (prevents division by zero).

The hybrid loss simultaneously optimizes region‐level overlap and pixelwise prediction accuracy, thereby improving segmentation robustness under class imbalance and heterogeneous tissue morphology.

### 3.4. Dual‐Stream Feature Extraction

Given a segmented histopathological patch *x*_*i*_, the corresponding tumor mask *m*_*i*_ is applied to preserve only the tumor region. Two complementary feature streams such as handcrafted radiomic descriptors and deep semantic embeddings are subsequently extracted. Radiomic features provide interpretable quantitative biomarkers describing tissue morphology, whereas texture and deep embeddings capture high‐level contextual representations. Their combination enables comprehensive characterization of IDC tissue for subsequent graph‐based learning.

#### 3.4.1. Radiomic Features

Radiomic features represent encoded quantitative tissue information and were extracted from tumor‐masked regions only. This consists of first‐order intensity measures (mean, variance, and skewness), texture features (entropy and contrast), and shape‐based features (area, perimeter, compactness, and solidity). These interpretable biomarkers represent morphology and textural heterogeneity of IDC tissue.

a. First‐order intensity statistics: captures image intensity information alone. The mean intensity *μ* and variance *σ* ^2^ are considered as first‐order features for computing, as shown in Equations (5) and (6) below.

(5) μ=1M∑p∈MIp,

(6) σ2=1M∑p∈MIp−μ2,

where *I*_*p*_ is the intensity value of a pixel at location *p* and *M* is a set of pixels defined by the tumor mask. These features attempt to capture global intensity related changes such as cellularity and staining intensity.

b. Texture features: Texture features extract the spatial relationship between pixel intensities using gray‐level co‐occurrence matrix (GLCM). If *P*(*i*, *j*) is the normalized GLCM showing the joint probability of pixel pair with intensities *i* and *j*, texture features are calculated using Equations (7) and (8) below.

(7) Entropy=∑gPglogPg,

(8) Contrast=∑i,ji−j2Pi,j,

where *P*_*g*_ is the probability of gray level *g*.

Entropy evaluates the randomness and heterogeneity of texture, whereas contrast indicates the variation in local intensity. Both these features signify the aggressiveness and disorganization of IDC tumor.

c. Morphological descriptors: Shape descriptors such as area, perimeter, solidity and compactness describe the shape of the tumor region. Irregularity, invasiveness, and deformation of tumors can be measured by these shape descriptors, which are also indications of malignancy.

Collectively, the extracted radiomic features form a vector *r*_*i*_ ∈ *ℝ* ^*m*^, providing an interpretable, quantitative representation of tissue morphology.

#### 3.4.2. Deep Features

Deep semantic features are extracted using a ResNet50 network pretrained on ImageNet and fine‐tuned for histopathological image analysis. The residual learning architecture facilitates effective optimization of deep networks through identity shortcut connections. For an input feature map x, the residual block output is computed as Equation (9), followed by ReLU activation (Equation 10). Feature vectors obtained from the global average pooling layer are used as the deep representation of each tumor patch.

(9) y=Fx,Wi+x,

where *F*(*x*, *W*_*i*_) denotes the residual function parameterized by learnable weights *W*_*i*_ and *x* represents the identity shortcut connection.

(10) h=σy,

where *σ*(·) denotes the ReLU activation function.

In the proposed model, feature vectors extracted from the final global average pooling layer of ResNet50 are used as deep semantic representations of the segmented tumor patches. These features capture high‐level contextual and structural characteristics of breast tissue and are subsequently fused with radiomic descriptors to form the hybrid deep‐radiomic feature representation.

#### 3.4.3. Hybrid Feature Fusion

To leverage the complementary strengths of handcrafted and learned representations, the radiomic feature vector *r*_*i*_ and deep embedding *d*_*i*_ are concatenated to form a unified hybrid feature vector and is represented in Equation (11) below.

(11) zi=ridi∈ℝp.

The concatenated feature vector is first L2‐normalized and subsequently projected onto a lower dimensional subspace using principal component analysis (PCA), yielding the compact hybrid representation as shown in Equation (12).

(12) zi′=PCAzizi2∈ℝp′.

This representation contains both low‐level histological statistics and high‐level semantic information that will be used to build the graph and perform contextual learning for IDC aggressiveness grading in the next stage.

### 3.5. Feature Fusion and Graph Construction

Following patch‐level feature fusion, a graph‐based representation is constructed to model the spatial and morphological relationships among histopathological tissue patches within a WSI. Unlike independent patchwise analysis, graph modeling preserves contextual interactions between neighboring and morphologically related tissue regions, which are essential for accurately assessing IDC aggressiveness. Each WSI is represented as an undirected weighted graph, as defined in Equation (13).

(13) G=V,E,

where *V* denotes the set of nodes corresponding to image patches and *E* represents the set of edges encoding interpatch relationships.

#### 3.5.1. Nodes Representation

Each node *v*_*i*_ is assigned the hybrid feature vector *z*_*i*_ obtained from Equation (11). For a WSI consisting of *N* patches, the complete node feature matrix is defined in Equation (14) below.

(14) Z=z1,z2,⋯,zN⊤.

This matrix provides a compact yet expressive representation of tissue morphology across the entire slide.

#### 3.5.2. Edge Construction

Graph connectivity is established using two complementary criteria such as spatial proximity and feature similarity. Spatial edges preserve local neighborhood relationships, whereas feature‐based edges capture long‐range morphological similarities.

a. Spatial proximity‐based edges

Spatial relations between patches are modeled as a function of Euclidean distance between patch centroid coordinates expressed in slide space. Let *p*_*i*_ = (*u*_*i*_, *v*_*i*_) represent the spatial coordinate location of patch *p*_*i*_. Two nodes are connected if their Euclidean distance satisfies the Equation (15).

(15) pi−pj2≤τ,

where *τ* is a distance threshold that sets the spatial neighborhood radius. The spatial proximity or adjacency matrix *A*_*s*_ is shown in Equation (16).

(16) Asi,j=exp−pi−pj222σp2, if pi−pj2≤τ0,           otherwise,

where *σ*_*p*_ determines how fast influence decreases spatially. This operation allows patches that are closer to each other to have higher weights and aggregate local context.

b. Feature similarity‐based edges

To capture long‐range morphological similarity, feature‐based edges are established using cosine similarity between hybrid node features as shown in Equation (17).

(17) si,j=zi·zjzi2 zj2,

where z_*i*_ and z_*j*_ are the hybrid deep‐radiomic feature vectors of nodes *v*_*i*_ and *v*_*j*_, respectively. ‖·‖_2 denotes the Euclidean (L2) norm.

An edge is created between nodes *v*_*i*_and *v*_*j*_ if the below mentioned condition of Equation (18) is satisfied.

(18) j∈Nki.

The resulting feature similarity adjacency matrix *A*_*f*_ is defined as shown in Equation (19) below.

(19) Afi,j=si,j,if j∈Nki0,otherwise.

The spatial and feature‐based adjacency matrices capture complementary aspects of interpatch relationships.

c. Composite edge weighting

To jointly incorporate spatial proximity and feature similarity, a composite edge weighting scheme is employed. The final edge weight *w*_*i* *j*_ between nodes *v*_*i*_ and *v*_*j*_ is defined in Equation (20) below.

(20) wij=exp−pi−pj222σp2.expsi,j−1T,

where *σ*_*p*_ regulates the influence of spatial distance, *s*(*i*, *j*) is cosine feature similarity, and *T* is a temperature parameter that controls the contribution of feature similarity. The resulting edge weights are normalized to the interval [0, 1], assigning larger weights to patches that are both spatially adjacent and morphologically similar. Thus, the resulting weighted graph jointly represents local tissue architecture and longer range morphological relationships.

The complete procedure for constructing the weighted hybrid deep‐radiomic graph and generating the slide‐level IDC aggressiveness prediction is summarized in Algorithm 1.

**Algorithm 1: Hybrid deep‐radiomic graph construction and contextual learning.**
*Input:*
 *WSI* *I*, *segmented tumor regions, spatial threshold* *τ* *, number of nearest neighbors* *k*
*Output:*
 *Slide-level IDC aggressiveness prediction* $\overset{\wedge }{y}$
1: *Extract tumor patches* *X* = {*x*_1_, *x*_2,_ ⋯ ⋯⋯⋯, *x*_*N*_} *from the segmented WSI.*
2: *For each patch* *x*_*i*_ *ϵ* *X* *, do*
3: *Extract deep representation* *d*_*i*_ *and radiomic representation* *r*_*i*_
4: *Form hybrid representation* *z*_*i*_⟵[*r*_*i*_‖*d*_*i*_]
5*: Normalize and reduce* *z*_*i*_ *to obtain* ${z_{i}}^{\text{’}}$.
6: *Define node* $v_{i}⟵{z_{i}}^{\text{’}}$ *and retain patch centroid* *p*_*i*_
7: *End For*
8: *Initialize graph* *G* = (*V*, *E*)
9: *For each node* *v*_*i*_, *do*
10: *Determine spatial neighbors using* ${\left\|\|p_{i}-p_{j}\right\|}_{2}\leq \tau$
11: *Determine feature neighbors* *N*_*k*_(i) *using cosine similarity.*
12: *For each connected pair* (*v*_*i*_, *v*_*j*_), *do*
13: *Compute edge weight* *w*_*i* *j*_ *using the joint spatial–feature weighting function defined*
   *in Equation (*(*20*))
14: *End For*
16: *End For*
15: *Construct the weighted graph* *G* = (*V*, *E*, *W*)
17: *Apply GAT to* *G* *to obtain contextual node representations* *H*
18: *Aggregate* *H* *using attention-based MIL to obtain slide representation* *h*_*s*_
19: *Predict IDC aggressiveness*: $\overset{\wedge }{y}⟵\text{Softmax}\left(Classifier\;\left(h_{s}\right)\right)$
20: *Return* $\overset{\wedge }{y}$

### 3.6. GAT‐Based GNN With MIL

The fused patch representations are organized as a graph and processed using a GAT to model contextual dependencies among neighboring tissue regions. By aggregating information from spatially adjacent and morphologically similar patches, the GAT learns context‐aware node representations that capture both local tissue architecture and global spatial organization. Because WSIs are annotated only at the slide level, an attention‐MIL framework is employed to aggregate patch embeddings into a discriminative slide‐level representation for IDC aggressiveness grading under weak supervision.

#### 3.6.1. Graph Attention–Based Contextual Learning

Given a graph *G* = (*V*, *E*), where each node *v*_*i*_ ∈ *V* is represented by the fused hybrid feature vector *h*_*i*_. The fused node feature vector *z*_*i*_ obtained from the graph construction stage is used as the initial node embedding ($h_{i}^{\left(0\right)}=z_{i}$) for GAT learning. A shared linear transformation is first applied to each node feature representation as shown in Equation (21).

(21) hi′=Whi,

where *W* denotes a learnable weight matrix and $h_{i}^{\prime }\;$represents the transformed node feature.

The importance of a neighboring node *j* with respect to node *i* is quantified through an attention mechanism. The unnormalized attention coefficient is computed as shown in Equation (22).

(22) eij=LeakyReLUaTWhiWhj

where *a* is a learnable attention vector and ∥ denotes vector concatenation.

The attention coefficients are subsequently normalized using the softmax function to obtain the relative contribution of each neighboring node as shown in Equation (23).

(23) αij=expeij∑k∈Niexpeik,

where $N\left(i\right)$ represents the set of neighboring nodes connected to node *i*.

The updated node representation is then obtained through weighted neighborhood aggregation as shown in Equation (24).

(24) hil+1=σ∑j∈NiαijWhj,

where *σ*(·) denotes a nonlinear activation function and *l* represents the current GAT layer.

Successive GAT layers progressively incorporate contextual information from spatially adjacent and morphologically similar tissue regions. This enables the framework to capture both local tissue architecture and global contextual dependencies, thereby improving the discriminative representation learning required for accurate IDC aggressiveness grading

#### 3.6.2. Attention‐Based Instance Weighting

To quantify the relative importance of each patch embedding *h*_*i*_, a learnable attention mechanism is applied. First, a latent attention representation is computed as shown in Equation (25) below.

(25) ui=tanhWuhi+bu,

where *h*_*i*_ ∈ *ℝ* ^*d*^ denotes the embedding of the *i*‐th patch and *W*_*u*_ and *b*_*u*_ are trainable parameters. The nonlinear activation stabilizes attention learning and enhances representational capacity. The attention weight *α*_*i*_ for each patch is then obtained using softmax normalization as represented in Equation (26) below.

(26) αi=expw⊤ui∑jexpw⊤uj,

where u_*i*_ is the transformed feature representation of patch *i*, w is the learnable attention vector, and *N* is the total number of patches (or instances) in the slide. The attention weights satisfy ∑_*i*_ *α*_*i*_ = 1. This formulation enables the model to automatically highlight discriminative tumor regions while downweighting irrelevant or redundant patches.

#### 3.6.3. Slide‐Level Aggregation and Classification

The integration of graph‐based contextual learning with attention‐based MIL enables robust weakly supervised slide‐level inference by jointly modeling spatial tissue organization and patch importance. Furthermore, the learned attention weights provide intrinsic explainability by identifying tissue regions that contribute most significantly to the final prediction. The slide‐level representation is computed as a weighted aggregation of patch embeddings as shown in below Equation (27).

(27) Hslide=∑iαi hi,

where *H*_slide_ captures the most informative morphological and contextual characteristics of the entire WSI. The aggregated representation is passed through a fully connected layer followed by a softmax classifier to predict the slide‐level IDC aggressiveness grade (low, intermediate, or high) as shown in Equation (28) below. The attention‐based MIL [49] aggregation is illustrated in Figure 2.

(28) y∧=softmaxFCHslide.

**Figure 2:**
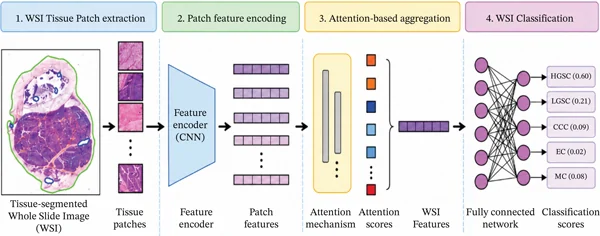
Attention‐based MIL.

### 3.7. Explainability Framework

The proposed model incorporates multiple explainability mechanisms to enhance transparency, trustworthiness, and clinical explainability. Multilevel explainability features would help understand decisions made by the model at both patch and slide levels.

• Grad‐CAM [50] is applied to the deep feature extraction network to generate patch‐level saliency maps, highlighting the histopathological regions that contribute most significantly to feature learning and IDC aggressiveness prediction.
• GAT attention and MIL attention maps provide visualization of the relative importance of tissue patches during graph aggregation and slide‐level prediction. These attention maps reveal how contextual relationships between neighboring tissue regions influence the final grading decision.
• SHAP [13] is employed to quantify the contribution of individual radiomic features to the final prediction, enabling feature‐level interpretation of the learned hybrid representation.

Together, these complementary explainability techniques provide visual, contextual, and quantitative insights into the model′s decision‐making process, thereby improving the transparency and clinical explainability of the developed framework.

## 4. Results and Discussion

### 4.1. Experimental Datasets

The proposed explainable hybrid deep‐radiomic graph learning framework was developed and evaluated using two publicly available benchmark histopathology datasets, namely the BACH and the BreakHis datasets. These datasets are widely adopted in computational pathology research for the development and benchmarking of automated breast cancer diagnosis and grading algorithms. These datasets were collected, anonymized, and released by their respective authors in accordance with applicable ethical guidelines and institutional approvals. The present study involves only secondary analysis of publicly available, fully deidentified data and does not involve direct patient recruitment, intervention, or access to identifiable patient information. Hence, additional institutional ethical approval and informed consent were not required. The detailed description of BACH and BreakHis datasets are presented below.

#### 4.1.1. BACH Dataset

The BACH dataset consists of 400 breast histopathology images (2048 × 1536 pixels) stained with H&E and scanned at 200× magnification, which are collected from four diagnosis classes, normal, benign, in situ, and invasive carcinoma with 100 images for each class. Because the dataset has an equal number of examples across four classes and pixel‐level annotations created by experts, it is widely used to benchmark AI algorithms developed for breast cancer histopathological image classification. This dataset is released by the organizers of BACH Grand Challenge and consists of anonymized and deidentified images only.

#### 4.1.2. BreakHis Dataset

BreakHis is an open‐source database of histopathological breast cancer images with a total of 7909 microscopic images captured using surgical biopsy from 82 patients. The biopsy samples were scanned at four optical magnification factors 40×, 100×, 200×, and 400× and consisted of both benign and malignant tumor cases. There are a total of 2480 benign images and 5429 malignant images of different histological subtypes of breast tumors available in the BreakHis dataset. This dataset provides wide variability in tissue morphology, stain color, and imaging conditions across different magnification factors.

### 4.2. Experimental Setup

The proposed architecture was implemented in Python 3.10 and PyTorch deep learning library. Experiments were performed using a workstation with NVIDIA RTX 4090 GPU (memory of 24 GB), Intel Core i9 CPU, and 64 GB RAM running Windows 11. The network was optimized using the Adam optimizer with an initial learning rate of 1 × 10^−4^, *β*_1_ = 0.9, *β*_2_ = 0.999, and a weight decay of 1 × 10^−5^. Cosine annealing learning rate scheduler was used during training for faster convergence. Network was trained for 100 epochs with batch size of 16. Early stopping patience of 15 epochs was used during training. Dropout was used during training with probability of 0.5 for regularization of fully connected layers. Grid‐search on the validation dataset was used to choose hyperparameters that resulted in best validation accuracy. Each image patch was modeled as a graph node with edges determined based on spatial connectivity and feature similarity. GAT50 used eight attention heads, and dimensionality of hidden features was set to 128 with output embedding of Size 64. Attention‐based MIL was used to aggregate patch‐level embeddings into slide‐level embeddings, which were used for final grading of IDC aggressiveness. Detailed implementation settings used in our experiments are listed in Table 3.

**Table 3:** Hyperparameter settings and implementation details of the proposed framework.

Hyperparameters	Values
Programming language	Python 3.10
Deep learning framework	PyTorch
GPU	NVIDIA RTX 4090 (24 GB)
CPU	Intel Core i9
RAM	64 GB
Optimizer	Adam
Initial learning rate	1 × 10^−4^
Learning rate scheduler	Cosine annealing
Pretraining strategy	Contrastive self‐supervised learning
Fine‐tuning strategy	Supervised learning
Batch size	16
Number of epochs	100
Early stopping	Patience = 15
Weight decay	1 × 10^−5^
Dropout	0.5
Activation function	ReLU
Classification loss	Crossentropy loss
GAT attention heads	8
Hidden feature dimension	128
Output embedding dimension	64
Graph construction	Spatial proximity + cosine feature similarity
Hyperparameter tuning	Grid‐search using validation set
Trainable parameters	29.4 million
Average training time	4.8 h (100 epochs) measured on RTX 4090 GPU
Average inference time	1.39 s/WSI
Peak GPU memory utilization	5.1 GB

The proposed framework adopts a two‐stage optimization strategy to achieve robust feature learning and reliable slide‐level IDC aggressiveness grading. In the first stage, the patch‐level feature encoder is pretrained using contrastive self‐supervised learning on unlabeled histopathological patches to learn discriminative tissue representations. In the second stage, the pretrained encoder is fine‐tuned using labeled training samples under supervised learning, enabling the extraction of task‐specific features for accurate aggressiveness classification. During supervised training, the network is optimized using the crossentropy loss, which minimizes the discrepancy between the predicted class probabilities and the corresponding ground‐truth IDC aggressiveness labels.

In addition to the hyperparameter configuration, Table 3 reports the average training time, inference time, peak GPU memory utilization, and number of trainable parameters to provide a comprehensive assessment of the computational requirements of the proposed framework. The model comprises approximately 29.4 million trainable parameters, achieves an average inference time of 1.39 s per WSI, and requires 5.1 GB of peak GPU memory. These results indicate a favorable trade‐off between predictive performance and computational efficiency despite the integration of segmentation, hybrid feature fusion, graph attention learning, and MIL. The reported classification results correspond to the average performance obtained over the BreakHis and BACH datasets using stratified fivefold crossvalidation, thereby providing a robust and unbiased estimate of the proposed framework′s generalization performance, maintaining patient‐level independence to prevent data leakage. These implementation settings ensure reproducibility of the proposed hybrid deep‐radiomic model. The implementation settings summarized in Table 3 ensure reproducibility of the proposed hybrid deep‐radiomic model.

The performance of the proposed architecture was evaluated using multiple complementary metrics to comprehensively assess segmentation accuracy, classification performance, calibration, and generalization capability. Segmentation performance was measured using the Dice coefficient and intersection over union (IoU). Classification performance was evaluated using overall accuracy, precision, recall, macro‐F1 score, ROC‐AUC, and Cohen′s *κ* statistic. Model calibration was assessed using the Brier score and reliability diagrams, whereas crossdataset evaluation was performed to examine the generalization capability of the proposed model.

### 4.3. Tumor Segmentation Performance

Accurate tumor segmentation is a prerequisite for reliable extraction of deep and radiomic features, thereby directly influencing the performance of downstream IDC aggressiveness grading. Table 4 compares the proposed Attention U‐Net with DenseCRF refinement against representative baseline segmentation models on the BreakHis and BACH datasets. The statistical analysis demonstrates that the proposed method consistently improves segmentation performance over all baseline methods. The proposed segmentation framework achieves the highest Dice coefficient (0.932) and IoU (0.879), representing improvements of 6.1% and 9.8%, respectively, over the baseline U‐Net. Likewise, the precision (0.921) and recall (0.907) values indicate effective suppression of both false‐positive and false‐negative predictions, demonstrating robust localization of tumor regions across heterogeneous tissue structures.

**Table 4:** Quantitative comparison of tumor segmentation performance on BreakHis and BACH datasets.

Model	Dice ↑	IoU ↑	Precision ↑	Recall ↑
U‐Net	0.871	0.781	0.862	0.845
DeepLabv3+	0.894	0.812	0.879	0.861
Proposed model	0.932	0.879	0.921	0.907

The visual comparison of tumor segmentation performance across different models is illustrated in Figure 3. The superior performance of the proposed framework is primarily attributed to the attention mechanism, which emphasizes diagnostically relevant histopathological regions while suppressing irrelevant background tissue. In addition, DenseCRF refinement preserves boundary continuity and reduces isolated segmentation errors, resulting in anatomically coherent tumor masks. Consequently, the improved segmentation quality provides a reliable foundation for subsequent radiomic feature extraction, graph‐based contextual learning, and accurate IDC aggressiveness grading.

**Figure 3:**
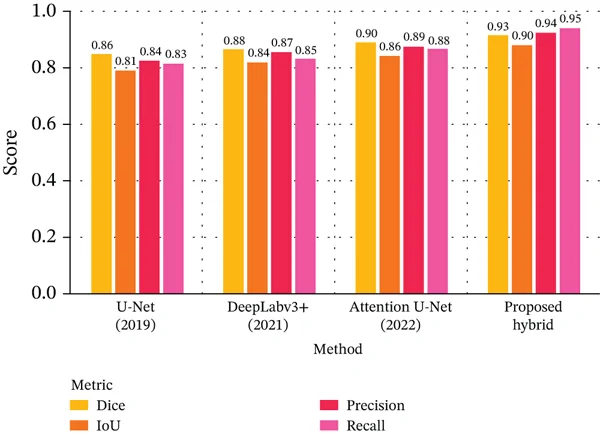
Visual comparison of tumor segmentation performance across different models.

### 4.4. IDC Aggressiveness Classification Performance

The classification performance of the proposed hybrid deep‐radiomic framework for multiclass IDC aggressiveness grading in comparison with representative baseline methods is demonstrated in Table 5.

**Table 5:** Classification performance for IDC grading.

Method	Accuracy ↑	Macro‐F1 ↑	ROC‐AUC ↑	*κ*‐score ↑
ResNet50	0.91	0.88	0.93	0.84
GNN‐MIL	0.93	0.91	0.95	0.87
Radiomics‐only SVM	0.89	0.86	0.92	0.82
Proposed model	0.98	0.968	0.98	0.94

In this study, the low‐, intermediate‐, and high‐aggressiveness categories were established by the research team using predefined histopathological image characteristics and experimental classification criteria. The assigned categories were subsequently reviewed and verified by pathologists to ensure consistency with the observed tissue morphology. We clarify that these are study‐specific classification categories and are not direct equivalents of the original diagnostic labels supplied with the BACH and BreakHis datasets.

The proposed method achieves better performance than all baseline approaches across the evaluated metrics, reaching an accuracy of 0.98, a macro‐F1 score of 0.968, a ROC‐AUC of 0.98, and a Cohen′s *κ* score of 0.94, demonstrating excellent discrimination ability and substantial agreement with the ground‐truth annotations. When compared with ResNet50, GNN‐MIL, and radiomics‐only approaches, our proposed architecture yields higher classification performance scores, validating the benefit of fusing complementary feature representations with graph‐based contextual learning.

To further establish the competitiveness of the proposed framework, its performance was compared with representative recent state‐of‐the‐art breast histopathology methods published between 2023–2025 and is presented in Table 6. Wherever available, comparisons were made using the same publicly available datasets and reported evaluation metrics. Because the existing studies differ in task formulation, dataset usage, and evaluation protocols, the results are interpreted as a comparative benchmark rather than a strictly controlled head‐to‐head comparison. The comparison includes studies evaluated on the BreakHis and/or BACH datasets and considers accuracy, F1‐score, AUC, and Dice coefficient, where these metrics were reported. The proposed framework achieves an accuracy of 98.30%, F1‐score of 0.968, AUC of 0.98, and Dice coefficient of 0.91. Its classification accuracy is comparable with or higher than that reported by recent methods, including Alotaibi et al. [22] (98.17%) and Zhou et al. [51] on BreakHis (98.22%). It also exceeds the reported accuracy of Venkat Gella [52] (97.34%) and Thatha et al. [33] (95.34%). These results demonstrate the competitive performance of the proposed framework while simultaneously integrating attention‐guided tumor segmentation, deep‐radiomic feature fusion, graph‐based contextual learning, and attention‐based MIL within a unified framework and is represented in Figures 4 and 5 below.

**Table 6:** Quantitative comparative performance of the proposed framework with state‐of‐the‐art methods.

Method	Dataset	Accuracy (%)	Macro‐F1	AUC	Dice
Alotaibi et al. [22]	BreakHis	98.17	0.961	0.97	0.89
Srikantamurthy et al. [31]	BreakHis	92.50	0.931	0.91	0.84
Thatha et al. [33]	BreakHis + BACH	95.34	0.946	0.93	0.85
Fiaz et al. [53]	BACH	94.00	0.964	0.92	0.85
Fiaz et al. [53]	Break His	95.29	0.936	0.93	0.84
Venkat Gella [52]	BreakHis + BACH	97.34	0.956	0.94	0.89
Zhou et al. [51]	BACH	96.40	0.963	0.98	0.88
Zhou et al. [51]	BreakHis	98.22	0.962	0.94	0.90
Proposed model	BreakHis + BACH	98.30	0.968	0.98	0.932

**Figure 4:**
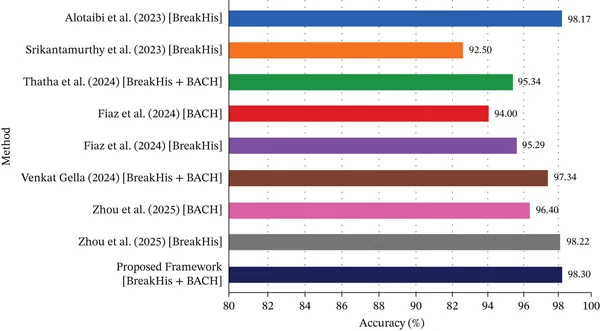
Comparison of classification accuracy of the proposed framework with recent state‐of‐the‐art methods.

**Figure 5:**
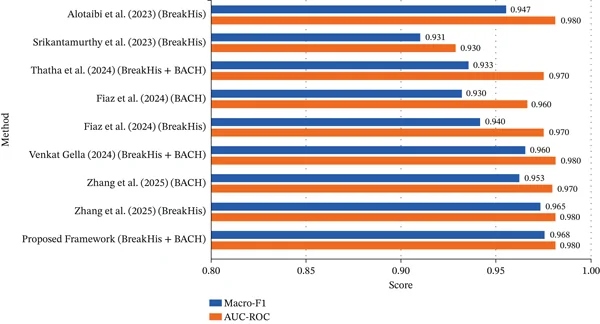
Comparative performance of recent state‐of‐the‐art methods in terms of Macro‐F1 and ROC‐AUC.

The improved performance can be attributed to the synergistic integration of attention‐guided tumor segmentation, complementary deep and radiomic feature extraction, attention‐based feature fusion, GAT‐based graph learning, and attention‐based MIL aggregation. Unlike conventional CNN‐ or transformer‐based approaches that primarily rely on learned deep representations, the proposed framework combines complementary morphological, radiomic, and contextual tissue characteristics to provide a more comprehensive representation of tumor heterogeneity. Furthermore, the integration of Grad‐CAM, SHAP, and graph attention visualizations provides multilevel model explainability, enabling interpretation at the image, feature, and graph levels. Collectively, these components contribute to improved classification performance, robust tissue representation, enhanced segmentation quality, and clinically interpretable IDC aggressiveness grading.

It should be noted that the results reported by existing studies were obtained using different experimental protocols, dataset partitions, classification settings, and evaluation criteria. Therefore, the numerical comparison should not be interpreted as a strictly controlled head‐to‐head benchmark. Nevertheless, it provides an informative assessment of the competitiveness of the proposed framework relative to recent breast histopathology methods evaluated on the same or related public datasets. Although Table 6 provides a quantitative comparison of predictive performance, numerical results alone do not fully explain the architectural factors contributing to the observed performance. Therefore, Table 7 presents a qualitative comparison of the proposed framework with recent state‐of‐the‐art methods, highlighting differences in deep feature extraction, radiomic feature integration, graph‐based learning, attention‐based MIL, explainability, and multiclass classification capabilities.

**Table 7:** Qualitative comparative performance of the proposed framework with state‐of‐the‐art methods.

Method	Deep features	Radiomics	Graph learning	MIL	Explainability	Multiclass
Alotaibi et al. [22]	✓	✗	✗	✗	✗	✓
Srikantamurthy et al. [31]	✓	✗	✗	✗	✗	✓
Thatha et al. [33]	✓	✓	✗	✗	✗	✓
Fiaz et al. [53]	✓	✗	✗	✗	✗	✓
Venkat Gella [52]	✓	✗	✗	✗	✗	✓
Zhou et al. [51]	✓	✗	✓	✓	Partial	✓
Proposed model	✓	✓	✓	✓	✓	✓

The comparison in Table 7 is based on key methodological characteristics, including deep feature extraction, radiomic feature integration, graph learning, attention‐based MIL, explainability, and multiclass IDC aggressiveness grading. Although recent approaches have demonstrated significant improvements in breast cancer classification using CNN, vision transformers, and hybrid deep learning architectures, most of them primarily rely on deep visual representations and do not simultaneously exploit complementary radiomic features, graph‐based contextual learning, and attention‐driven slide‐level aggregation. The proposed framework distinguishes itself by integrating attention‐guided tumor segmentation, hybrid deep and radiomic feature extraction, GAT‐based GNN, attention‐based MIL, and multilevel explainability within a unified end‐to‐end architecture. Unlike existing methods, which generally focus on either deep feature learning or transformer‐based representations, the proposed model effectively captures local morphological characteristics, quantitative radiomic biomarkers, and spatial contextual relationships among tissue regions while providing interpretable predictions through Grad‐CAM, SHAP, and graph attention visualizations. Furthermore, the framework supports multiclass IDC aggressiveness grading, making it more suitable for clinically relevant decision support. Overall, the qualitative comparison demonstrates that the proposed hybrid deep‐radiomic model offers a more comprehensive, interpretable, and clinically applicable solution than state‐of‐the‐art methods.

The complementary integration of deep learning, radiomics, graph learning, attention‐based MIL, and explainable AI enables the proposed framework to effectively model both local tissue morphology and global contextual relationships while maintaining clinical explainability. This comprehensive design addresses several limitations of existing state‐of‐the‐art methods and highlights the methodological advantages of the proposed framework, as summarized in Table 7.

To demonstrate the effectiveness and robustness of the proposed approach for automated IDC aggressiveness grading across different categories, the gradewise classification accuracy is presented in Table 8 and Figure 6.

**Table 8:** Gradewise classification performance comparison.

Grade	CNN	ResNet50	GNN‐MIL	Proposed hybrid
Low grade	0.87	0.90	0.92	0.94
Intermediate grade	0.82	0.85	0.90	0.92
High grade	0.89	0.92	0.94	0.96

**Figure 6:**
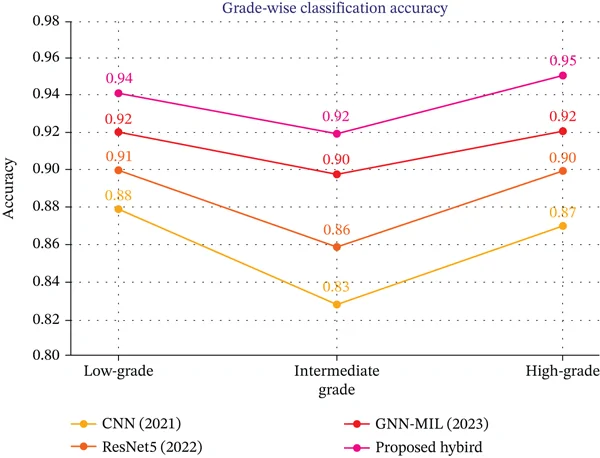
Gradewise classification accuracy.

The proposed hybrid deep‐radiomic model consistently achieves the highest accuracy across low‐, intermediate‐, and high‐grade IDC, with the greatest improvement observed for the intermediate‐grade category. This finding is particularly significant because intermediate‐grade lesions often exhibit substantial morphological overlap with adjacent grades, making them more challenging to classify than clearly low‐ or high‐aggressiveness lesions. The consistently high accuracy across all three grades indicates balanced performance with minimal interclass confusion, demonstrating that the proposed architecture effectively captures subtle morphological differences between adjacent aggressiveness categories.

Violin plots showing the prediction probability distributions of the three IDC aggressiveness grades are given in Figure 7. The separation between low‐grade, intermediate‐grade, and high‐grade class distributions indicates that the developed model learns separable features for each of the grades of IDC aggressiveness. Moreover, most of the prediction samples corresponding to correct predictions are concentrated in a narrow range, indicating a low level of prediction uncertainty. The less overlap between the distributions also indicates that proposed model learned to discriminate the morphological changes across the IDC grades, even when such changes are extremely subtle.

**Figure 7:**
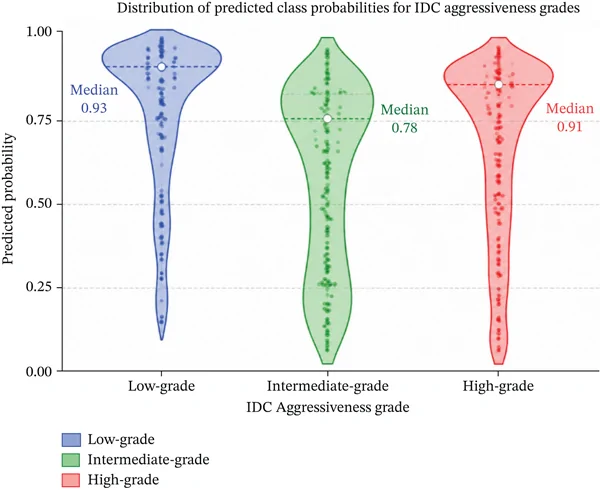
Violin plot illustrating the distribution of predicted class probabilities for low‐, intermediate‐, and high‐grade IDC categories.

Figure 8 shows a 2D scatter plot of the learned hybrid deep‐radiomic features embeddings extracted by the proposed approach using t‐SNE. Each cluster formed in the feature embedding space represents low‐, intermediate‐, and high‐grade IDC classes. We observe that these embeddings have learned morphological and textural representations that are unique to each IDC grade, with low‐, intermediate‐, and high‐grade clusters being well separated from each other demonstrating high interclass variance. Meanwhile, samples that belong to the same cluster are tightly grouped illustrating low intraclass variance. Additionally, as expected in the pathological scenario, theintermediate samples are learned to be closer to the boundary between low‐ and high‐grade embeddings. This is an intuitive result because intermediate‐grade indicates more complex pathological features of tumor aggressiveness when compared with low/high‐grades alone. This provides evidence that the proposed hybrid feature representation with graph‐based contextual learning is effective for IDC aggressiveness grading task.

**Figure 8:**
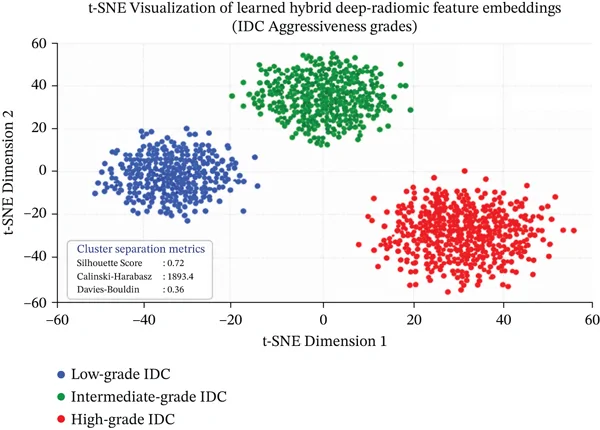
Two‐dimensional t‐SNE scatter plot of the learned hybrid deep‐radiomic feature embeddings.

Overall, the quantitative results, classwise analysis, and feature‐space visualizations consistently demonstrate that integrating deep semantic representations, handcrafted radiomic descriptors, graph‐based contextual learning, and attention‐based MIL enables more reliable and discriminative IDC aggressiveness grading than existing CNN‐ and graph‐based approaches.

### 4.5. Explainability and Feature Contribution Analysis

Beyond achieving high classification accuracy, clinical deployment requires transparent and interpretable decision‐making. Therefore, a comprehensive explainability analysis was performed using SHAP‐based radiomic feature attribution, Grad‐CAM visualizations, and GNN‐MIL attention maps to investigate the contribution of handcrafted features, deep representations, and graph‐based contextual reasoning to the final IDC aggressiveness prediction. SHAP‐based feature attribution analysis of Figure 9 reveals that features from texture filters overwhelmingly dominate aggressiveness grading decision with 43%, whereas features of morphological stream and intensity‐based stream take up 34.1% and 22.9%, respectively. Such results suggest that the learned representation highly relies on texture heterogeneity and structural irregularity to depict tumor aggressiveness, which agrees with the pathological intuition of IDC.

**Figure 9:**
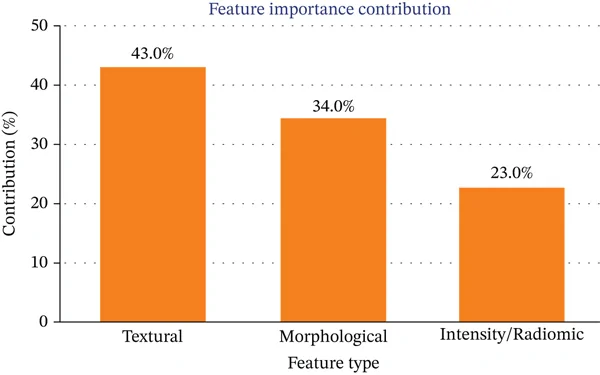
Feature importance contribution.

The visual explanations generated by Grad‐CAM and GNN‐MIL attention mechanisms are presented in Figures 10 and 11. Grad‐CAM highlights diagnostically relevant histopathological regions, including nuclei‐dense tumor areas, stromal structures, and regions exhibiting characteristic breast cancer progression patterns while suppressing irrelevant background tissue. Complementarily, the GNN and MIL attention maps identify the tissue patches that contribute most significantly to the slide‐level prediction, providing insight into the contextual reasoning learned by the graph‐based framework.

**Figure 10:**
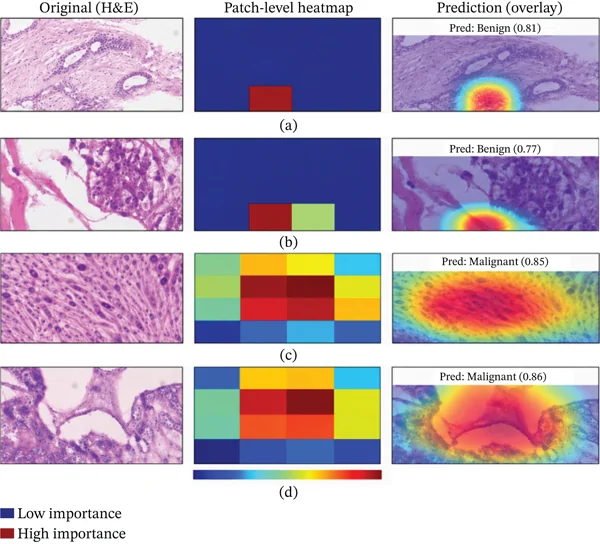
BreakHis dataset binary breast cancer classification using Grad‐CAM visualizations. Visual explanations generated by Grad‐CAM and GNN‐MIL attention mechanisms for representative histopathological samples: (a) benign sample with localized attention (prediction confidence: 0.81); (b) benign sample with attention over adjacent patches (0.77); (c) malignant sample with prominent activation across densely cellular tumor regions (0.85); and (d) malignant sample with attention around tumor‐associated glandular structures (0.86). Each row presents the original H&E image, patch‐level attention heatmap, and corresponding prediction overlay.

**Figure 11:**
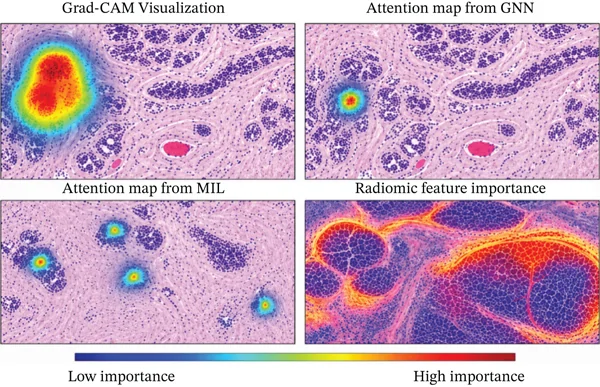
Multilevel explainability analysis of the proposed hybrid deep‐radiomic graph learning.

Table 9 summarizes the complementary explainability mechanisms incorporated into the proposed framework and their respective contributions toward clinical interpretability. By integrating complementary feature‐level, region‐level, and graph‐level explanations, the proposed framework provides a comprehensive interpretation of the decision‐making process, consequently improving transparency and facilitating clinical validation.

**Table 9:** Explainability and clinical transparency.

Explainer	Key output	Clinical usefulness	Performance
Grad‐CAM	Patch‐level tumor focuses heatmaps	Region‐level saliency	Strong
GNN attention	Slide‐level node‐importance map (*α*_i_)	Patch relevance ranking	Strong
SHAP radiomics	Quantitative contribution of radiomic features	Featurewise explainability	Strong
Proposed model	Unified multimodal explanations (Deep + radiomic + graph)	Highest transparency	Excellent

From a pathological perspective, the explainability results demonstrate that the proposed framework consistently focuses on histological characteristics routinely examined by breast pathologists during grading of IDC. Grad‐CAM visualizations predominantly highlight tumor‐rich regions exhibiting increased nuclear density, pleomorphism, irregular glandular architecture, and stromal invasion, all of which are recognized indicators of tumor aggressiveness. Similarly, the GNN and MIL attention maps emphasize spatially correlated tissue regions rather than isolated image patches, suggesting that the framework effectively captures the architectural organization of the tumor microenvironment. The SHAP‐based radiomic analysis further reveals that texture‐ and morphology‐related descriptors contribute more substantially to the final prediction than intensity‐based features. This observation is clinically meaningful because increased tissue heterogeneity, nuclear atypia, irregular gland formation, and disruption of stromal architecture are well‐established pathological hallmarks associated with higher histological grades and poorer prognosis in IDC. The close agreement between the model′s explanations and established pathological assessment criteria indicates that the proposed framework learns biologically relevant tissue characteristics rather than relying on spurious image artifacts. Consequently, the explainability mechanisms enhance transparency, improve clinician confidence in the automated predictions, and support the potential integration of the proposed framework as an interpretable decision support tool in digital pathology workflows.

### 4.6. Crossdataset Generalization and Calibration

Unlike the results presented in the previous sections, which are based on stratified fivefold crossvalidation on the combined dataset, this experiment evaluates the robustness and generalization capability of the proposed framework under an independent crossdataset validation protocol. The experiments were conducted by training the model on the BreakHis dataset and evaluating it on the independent BACH dataset, therefore assessing its ability to generalize across datasets acquired under different imaging conditions. The corresponding results are presented in Figure 12. Despite variations in staining protocols, image resolution, and acquisition conditions, the proposed framework consistently maintains good classification performance across both datasets, demonstrating strong robustness to domain shifts. These properties enable generalizability across datasets. The observed generalization can be attributed to three complementary factors: (i) stain normalization, which reduces interslide color variability; (ii) the hybrid deep‐radiomic representation, which captures both local cellular characteristics and global tissue morphology; and (iii) graph‐based contextual learning, which preserves spatial dependencies among neighboring tissue regions. Collectively, these components reduce dependence on dataset‐specific artifacts and improve robustness across different acquisition conditions.

**Figure 12:**
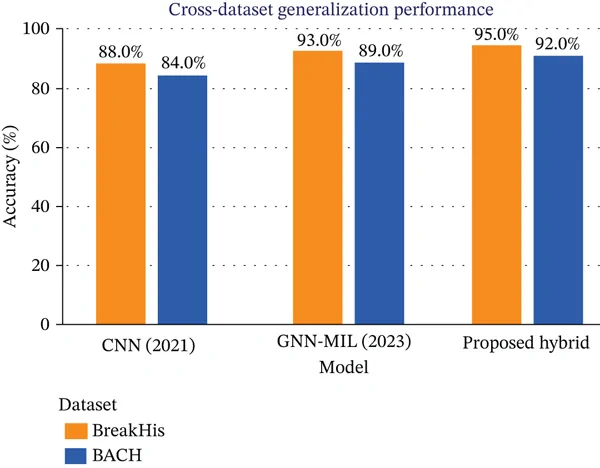
Crossdataset generalization performance.

Although crossdataset performance demonstrates the robustness of the learned representations, reliable probability estimation is equally important for clinical decision support. Therefore, the calibration characteristics of the proposed framework were also evaluated and represented in Table 10. The calibration results in achieving lowest Brier score of 0.041 with expected calibration error (ECE = 0.018) and negative log likelihood (NLL = 0.112) among the baseline models, suggesting that the predicted probabilities match with the true outcome frequencies well. Accurate calibration is particularly important in clinical decision support systems because confidence estimates are often used for treatment planning and risk stratification. The low calibration errors observed for the proposed framework suggest that highly confident predictions are more likely to be correct, consequently improving the reliability and clinical usability of automated IDC aggressiveness grading.

**Table 10:** Calibration and reliability measures.

Method	Brier score ↓	ECE ↓	NLL ↓
ResNet50	0.092	0.061	0.285
GNN‐MIL	0.074	0.048	0.201
Temperature‐scaled CNN	0.063	0.034	0.168
Proposed model	0.041	0.018	0.112

Overall, the crossdataset evaluation and calibration analysis demonstrate that the proposed framework not only generalizes effectively across heterogeneous histopathological datasets but also produces well‐calibrated confidence estimates, making it suitable for reliable clinical decision support.

### 4.7. Computational Efficiency and Complexity

Despite integrating attention‐guided segmentation, hybrid deep‐radiomic feature extraction, graph‐based contextual learning, and attention‐based MIL, the proposed framework maintains competitive computational efficiency. As illustrated in Figure 13, the computational statistics indicate that the proposed framework maintains moderate computational complexity despite integrating multiple processing modules. The framework achieves an effective balance between predictive performance and computational resource utilization, requiring an average inference time of 1.39 s per WSI with a peak GPU memory consumption of 5.1 GB. These results demonstrate the practical feasibility of deploying the proposed framework in large‐scale digital pathology applications.

**Figure 13:**
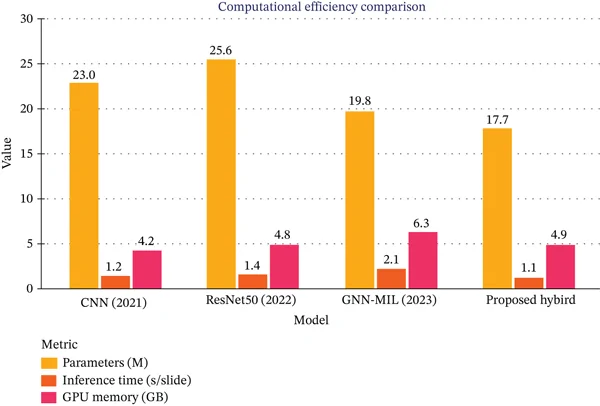
Computational efficiency comparison.

To provide a more comprehensive analysis of its computational characteristics, the theoretical time complexity and stagewise execution times are presented in Tables 11 and 12, respectively. The theoretical time‐complexity analysis presented in Table 11 demonstrates that the computational overhead introduced by the proposed architecture remains manageable. Both CNN‐based feature extraction and graph operations are responsible for the majority of the computational cost. On the other hand, radiomic feature extraction adds little extra overhead to this cost. Additionally, the slight increase in complexity is warranted by the gain in performance obtained by means of hybrid feature integration and contextual modeling.

**Table 11:** Time‐complexity comparison.

Model	Patch encoding	Radiomics/other	MIL/aggregation	Inference complexity (per slide)	Training (*S*, slides; *I* iters)
ResNet50 (CNN)	*O*(*N*.*C*_*C* *N* *N*_)	—	*O*(*N*.*d*)	*O*(*N*.*C*_*C* *N* *N*_ + *N*.*d*)	*O*(*I*.*S*.*N*.*C*_*C* *N* *N*_)
Radiomics‐only (SVM)	Optional/*0*	*O*(*N*.*R*)	*O*(*N*.*R*)	O(N·R) (+ CNN cost if used)	*O*(*M* ^2^.*C*_*k* *e* *r* *n* *a* *l*_)
GNN‐MIL	*O*(*N*.*C*_*C* *N* *N*_)	Optional *O*(*N*.*R*)	Attention‐based MIL *O*(*N*.*d*)	*O*(*N*.*C*_*C* *N* *N*_ + *N* *l* *o* *g* *N* + *L*_*g*_.*k*.*N*.*d*)	*O*(*I*.*S*.(*N*.*C*_*C* *N* *N*_ + *N* *l* *o* *g* *N* + *L*_*g*_.*k*.*N*.*d*)
Proposed model	*O*(*N*.*C*_*C* *N* *N*_)	*O*(*N*.*R*)	Attention‐based MIL *O*(*N*.*d*)	*O*(*N*.*C*_*C* *N* *N*_ + *N*.*R* + *N* *l* *o* *g* *N* + *L*_*g*_.*k*.*N*.*d*)	*O*(*I*.*S*.(*N*.*C*_*C* *N* *N*_ + *N*.*R* + *N* *l* *o* *g* *N* + *L*_*g*_.*k*.*N*.*d*)

**Table 12:** Average processing time per histopathological patch.

Processing stage	Average time (ms)
Attention U‐Net segmentation	8.4
DenseCRF refinement	3.1
Deep feature extraction (ResNet50)	5.6
Radiomic feature extraction	2.3
Hybrid feature fusion	0.4
Graph construction	1.2
GAT + MIL classification	2.8
Total processing time per patch	23.8 ms

The computational complexity analysis presented in above Table 11 uses the following notation. Let *N* denote the number of image patches (graph nodes) extracted from a WSI, *C*_*C* *N* *N*_ the computational cost of CNN‐based patch feature extraction, *R* the computational cost of radiomic feature extraction per patch, *d* the feature embedding dimension, *L*_*g*_ the number of GAT layers, *k* the average number of neighboring nodes connected to each graph node, *S* the total number of training slides, *I* the number of training iterations (epochs), and *M* the number of training samples used by the kernel‐based SVM classifier. During inference, the proposed framework first extracts deep and radiomic features from all patches with a computational cost of *O*(*N*.*C*_*C* *N* *N*_) and *O*(*N*.*R*), respectively. The graph is subsequently constructed with an approximate complexity of *O*(*N*log*N*), followed by GAT‐based graph representation learning requiring *O*(*L*_*g*_.*k*.*N*.*d*), where graph attention coefficients and neighborhood feature aggregation are computed across *L*_*g*_ layers. Finally, the attention‐based MIL module aggregates the node embeddings into a slide‐level representation with a linear complexity of *O*(*N*.*d*), followed by slide‐level classification. Consequently, the overall inference complexity of the proposed hybrid deep‐radiomic framework is *O*(*N*.*C*_*C* *N* *N*_ + *N*.*R* + *N*log*N* + *L*_*g*_.*k*.*N*.*d*), whereas the corresponding training complexity over *S* slides and *I* iterations is *O*(*I*.*S*(*N*.*C*_*C* *N* *N*_ + *N*.*R* + *N*log*N* + *L*_*g*_.*k*.*N*.*d*)).

In addition to the theoretical complexity analysis, the practical computational performance of the proposed framework was evaluated in terms of training time, inference speed, memory utilization, model complexity, and patch‐level processing time. Furthermore, the framework comprises approximately 29.4 million trainable parameters, reflecting a moderate level of model complexity despite integrating attention‐guided tumor segmentation, adaptive deep‐radiomic feature fusion, graph attention learning, and attention‐based MIL. This is presented in Table 12 below.

Table 12 reports the average execution time of each processing stage in the proposed pipeline. Attention U‐Net segmentation and deep feature extraction account for the largest proportion of the computational cost, whereas graph construction, hybrid feature fusion, and GAT‐MIL classification introduce comparatively lower overhead. The complete pipeline requires an average processing time of 23.8 ms/patch, corresponding to an effective throughput of approximately 42 patches/s.

Table 13 compares the computational performance of the proposed framework with representative recent methods. Although the proposed framework integrates multiple processing stages, including attention‐guided tumor segmentation, hybrid deep‐radiomic feature extraction, GAT‐based contextual learning, and attention‐based MIL, it maintains competitive computational efficiency. The proposed model requires an average training time of 4.8 h, an inference time of 1.39 s/WSI image, and an average processing time of 23.8 ms/histopathological patch while utilizing 5.1 GB of GPU memory. Although the proposed framework integrates multiple complementary processing modules, the associated computational overhead remains moderate and is offset by substantial improvements in segmentation quality, IDC aggressiveness grading, contextual representation learning, and multilevel explainability.

**Table 13:** Comparison of computational performance with recent methods.

Method	Training time	Testing time/WSI	Patch time	GPU	Parameters
ResNet50 (2022)	3.2 h	0.95 s	18.6 ms	RTX 3090	25.6 M
GNN‐MIL (2023)	4.1 h	1.21 s	21.5 ms	RTX 3090	27.9 M
Fiaz et al. [53]	5.4 h	1.84 s	NR	RTX 4090	32.8 M
Zhou et al. [51]	5.8 h	1.72 s	NR	RTX 4090	35.1 M
Proposed model	4.8 h	1.39 s	23.8 ms	RTX 4090	29.4 M

These results demonstrate a favorable trade‐off between predictive performance, computational efficiency, and practical deployability for large‐scale digital pathology workflows.

### 4.8. Statistical Significance and Crossvalidation Analysis

To quantitatively assess the stability and statistical significance of our framework, fivefold crossvalidation experiments were performed on the combined BreakHis and BACH datasets. Table 14, summarizes foldwise classification accuracy for the proposed framework and other baseline methods. The proposed framework obtains competitive classification results in all folds with mean accuracy of 98.30*%* ± 0.21*%*, which performs better compared with baseline models in terms of both mean accuracy and standard deviation. This result suggests that our model is stable and has small variance over different subsets of data. Furthermore, paired *t*‐test was applied to verify whether these improvements are statistically significant. The null hypothesis assumes that our proposed model performs equally compared with other baseline methods.

**Table 14:** Fivefold crossvalidation performance of the proposed framework and baseline models.

Method	Fold‐1 (%)	Fold‐2 (%)	Fold‐3 (%)	Fold‐4 (%)	Fold‐5 (%)	Mean ± SD (%)
ResNet50	90.8	91.4	91.0	91.8	91.1	91.22 ± 0.38
Radiomics‐only SVM	89.3	90.1	89.8	90.0	89.9	89.82 ± 0.33
GNN‐MIL	93.1	93.8	93.6	93.3	93.7	93.50 ± 0.29
Proposed model	98.0	98.4	98.5	98.2	98.4	98.30 ± 0.21

Further, Table 15 represents paired *t*‐test statistical validation of the proposed framework. It is observed that *p* values of all the paired tests are less than 0.05, implying that there is statistical significance at 95% confidence level. These findings demonstrate that the integrated hybrid deep‐radiomic graph learning framework consistently provides superior predictive performance compared with existing approaches.

**Table 15:** Paired *t*‐test statistical validation of the proposed framework.

Comparison	Mean difference (%)	*t* value	*p*	Statistical significance
Proposed vs. ResNet50	7.08	10.84	< 0.001	Significant
Proposed vs. radiomics‐only SVM	8.48	13.17	< 0.001	Significant
Proposed vs. GNN‐MIL	4.80	8.32	0.001	Significant

Overall, the crossvalidation and statistical significance analyses demonstrate that the proposed framework consistently achieves robust and reproducible performance across different data partitions, providing strong evidence for its reliability in automated IDC aggressiveness grading.

### 4.9. Ablation Study

To quantitatively measure the impact of each component, we performed componentwise ablation study by gradually adding each major module of the proposed framework under identical training, validation, and evaluation settings of training/validation and evaluation. Starting from CNN as baseline model, we studied the effects of Attention U‐Net, deep‐radiomic feature fusion, GAT and attention‐based MIL sequentially. The quantitative results are reported in Table 16, which show that model performance consistently improved with the addition of each building block.

**Table 16:** Componentwise ablation analysis of the proposed framework.

Configuration	Attention U‐net	Radiomics	GAT	MIL	Accuracy (%)	Macro‐F1	ROC‐AUC
Baseline CNN	✗	✗	✗	✗	90.2	0.86	0.92
+ Attention U‐Net	✓	✗	✗	✗	91.4	0.88	0.94
+ Deep‐radiomic fusion	✓	✓	✗	✗	93.6	0.92	0.95
+ GAT	✓	✓	✓	✗	95.2	0.94	0.97
Proposed model	✓	✓	✓	✓	98.3	0.968	0.98

The base CNN attained an accuracy of 90.2%, macro‐F1 score of 0.86, and ROC‐AUC of 0.92 suggesting that deep semantic features alone are capable of achieving moderate performance for IDC aggressiveness grading. Adding Attention U‐Net boosted accuracy to 91.4%, macro‐F1 score to 0.88, and ROC‐AUC to 0.94. Attention‐guided segmentation allows the network to spatially discount areas of nontumor tissue and focus on localizing clinically relevant tumor areas allowing for more robust feature extraction. With the addition of deep‐radiomic feature fusion, there was a significant boost in performance with an accuracy of 93.6%, macro‐F1 score of 0.92, and ROC‐AUC of 0.95. The radiomic performance gain reveals that handcrafted radiomic features contain complementary and useful quantitative information about tissue texture, morphology, and intensity that complements high‐level semantic features learned by the deep network. As a result, the adaptive fusion of both feature types leads to a more expressive representation of tumor heterogeneity. With the addition of GAT, classification performance was able to improve to an accuracy of 95.2%, macro‐F1 score of 0.94, and ROC‐AUC of 0.97. This gain demonstrates that modeling explicit spatial contextual relationships between neighboring tissue patches through assigning adaptive attention weights to neighboring nodes allows the GAT to learn heterogeneous tissue dependencies and tumor microenvironment properties that are not accessible in patchwise independent analysis. Lastly, we achieved the best performance when we applied attention‐based MIL. Our attention‐based MIL model reached 98.3% accuracy, 0.968 macro‐F1 score, and 0.98 ROC‐AUC. Attention‐based MIL allows for identification and emphasis of diagnostically relevant tissue patches while suppressing redundant noise patches during aggregation, allowing for more robust slide‐level representations to be learned. Overall, our framework outperformed our baseline CNN model by an absolute 8.1 percentage point increase in accuracy and also saw significant increases in macro‐F1 score (0.86–0.968) and ROC‐AUC (0.92–0.98). Because our framework saw consistent increases across all evaluation metrics with the addition of each architectural design, we can conclude that each component provides a benefit to the framework as a whole rather than giving an incremental performance improvement.

The ablation study confirms that each architectural component contributes positively to the overall framework. Attention U‐Net enhances tumor localization, hybrid deep‐radiomic feature fusion enriches tissue representation, GAT captures spatial contextual dependencies, and attention‐based MIL enables effective slide‐level aggregation. The cumulative improvement across all evaluation metrics demonstrates that these modules operate synergistically, resulting in a more accurate, robust, and clinically interpretable framework for automated IDC aggressiveness grading.

## 5. Conclusion and Future Work

This study presented an explainable hybrid deep‐radiomic graph learning framework for automated multiclass IDC aggressiveness grading from breast histopathology images. The framework integrates attention‐guided tumor segmentation with DenseCRF refinement, complementary deep and radiomic feature representations, graph‐based spatial contextual learning using GAT, and attention‐based MIL for slide‐level prediction. Multilevel explainability using Grad‐CAM, GNN attention, and SHAP further provides insight into the image regions, contextual relationships, and features contributing to model predictions. Experimental evaluation on the BACH and BreakHis datasets demonstrated that the proposed framework achieved 98.3% accuracy, 0.968 macro‐F1, 0.98 ROC‐AUC, and 0.94 Cohen′s *κ* while maintaining competitive computational requirements. The ablation and statistical analyses further demonstrated the contribution and robustness of the integrated framework, and the crossdataset evaluation provided evidence of its generalization capability. Overall, the results indicate that jointly modeling morphological, radiomic, and spatial contextual information can improve IDC aggressiveness grading compared with conventional deep learning and graph‐based approaches.

Despite these promising results, further validation on larger and multi‐institutional whole‐slide datasets is required to establish robustness across variations in staining, scanners, tissue preparation, and clinical populations. Future work will focus on external clinical validation, improved domain generalization, and further optimization of computational efficiency to facilitate practical deployment in digital pathology workflows.

## Funding

No funding was received for this manuscript.

## Conflicts of Interest

The authors declare no conflicts of interest.

## Data Availability

The data that support the findings of this study are openly available in Breast Cancer Histopathological Database (BreaKHis) at https://web.inf.ufpr.br/vri/databases/breast‐cancer‐histopathological‐database‐breakhis/, Reference Number 10.1109/TBME.2015.2496264.
